# Four years of natural progressive course: A rare case report of juvenile Xp11.2 translocations renal cell carcinoma with *TFE3* gene fusion

**DOI:** 10.1515/med-2024-0985

**Published:** 2024-06-29

**Authors:** Liang Hu, Lina Li, Angcheng Li, Jianyong Tong

**Affiliations:** Department of Urology, Affiliated Jinhua Hospital, Zhejiang University School of Medicine, Jinhua, China; College of Medicine, Jinhua Polytechnic, Jinhua, Zhejiang, China; Department of Pathology, Affiliated Jinhua Hospital, Zhejiang University School of Medicine, Jinhua, China

**Keywords:** renal cell carcinoma, Xp11.2 translocations, *TFE3*, radical nephrectomy, natural progressive course

## Abstract

**Background:**

Renal cell carcinoma (RCC) with *TFE3* gene fusion caused by Xp11.2 translocations is a rare RCC subtype. This tumor is typically seen in children, comprising 20‒40% of overall RCC cases compared to 1‒1.6% observed in adults. Xp11.2 RCC is associated with a poor prognosis due to both the progression of local lesions and early distant and lymphatic metastasis.

**Case presentation:**

A case of RCC with Xp11.2 RCC translocations and *TFE3* gene fusion was found in a pediatric patient, illustrating the catastrophic effects of ignoring the condition. The tumor developed from a local lesion to lymph metastasis (3.2–12 cm) within 4 years. Despite ongoing controversy, surgical resection remains the most common and productive approach. In this patient, renal retroperitoneal lymph node dissection and radical nephrectomy of the left kidney were performed via laparoscopic surgery. The RCC-associated Xp11.2 translocation/*TFE3* gene fusions were identified by postoperative pathology. Microscopic analysis showed the presence of intravascular cancer thrombus, renal sinus invasion, and cancer necrosis. The pathological stages were confirmed as PT3aN1M0 with a negative margin. Follow-up at 5 months showed that the patient recovered without the use of any adjuvant treatments.

**Conclusion:**

Our study highlights the natural course, diagnosis, and treatment of RCC-associated Xp11.2 translocation/TFE3 gene fusions, especially the necessity of early surgery. This case may be a helpful reference for urologists in the treatment of similar cases. It also serves as a precautionary signal for patients who neglect the renal neoplasm.

## 1 Introduction

Renal cell carcinoma (RCC) caused by Xp11.2 translocation/*TFE3* gene fusion is an uncommon RCC subtype that was first identified by the World Health Organization (WHO) in 2004 [1]. The prognosis of this tumor is worse than other RCC subtypes [2]. In 2016, the WHO introduced molecular-driven histotypes termed the MiT family, which comprises *TFE3*, MiTF, TFEB, and TFEC. Consequently, these tumors were identified as MiT-RCC, indicating miT family translocation RCC.

The tumor is more prevalent in children and teenagers compared to adults. The tumor differs from other RCC types in the presence of various translocations on chromosome Xp11.2, resulting in a gene fusion involving *TFE3*; there are at least six possible fusion types [3] which are currently described as *TFE3*-rearranged RCC, the predominant subtype. In addition, RCC associated with Xp11.2 translocations exhibits a more aggressive progression than other RCC subtypes, characterized by the rapid progression of local lesions and early distant and lymphatic metastases.

Moreover, the tumor’s resistance to chemotherapy and radiotherapy results in an ultimately poor prognosis [4]. Surgical resection is considered the most effective approach for managing Xp11.2-translocation RCC. The prognosis for pediatric patients is usually more favorable than that of adults.

The present study presents a rare case of a patient who developed Xp11.2-translocation RCC with *TFE3* gene fusion over 4 years. A review of the relevant literature is included. It is hoped that this will increase awareness of this uncommon and often deadly disease.

## 2 Case presentation

**Figure 1:**
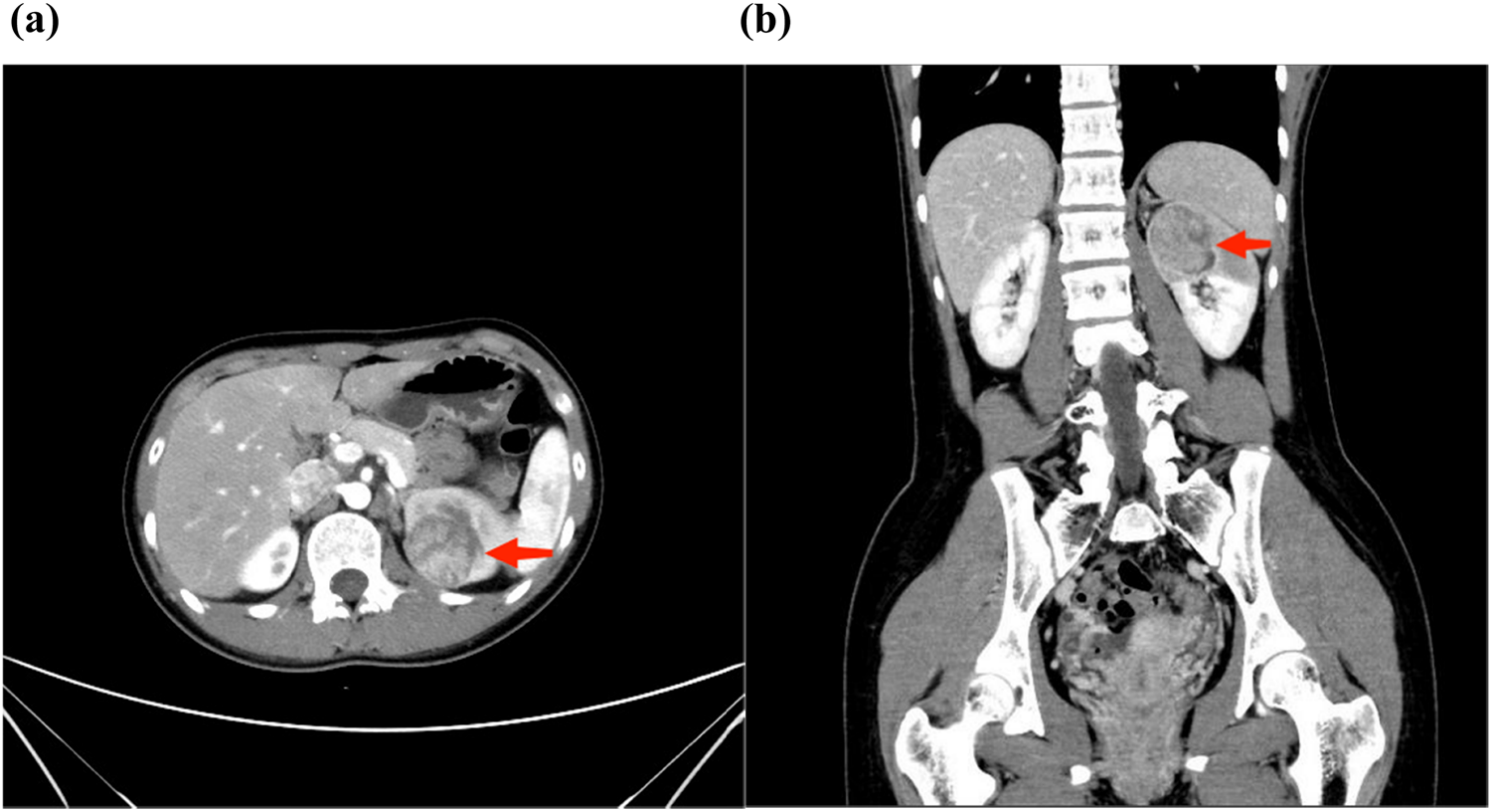
eCT image showing a solid mass (3.2 cm × 3.1 cm) in the upper pole of the left kidney in 2019: (a) transverse plane and (b) coronal plane.

**Figure 2:**
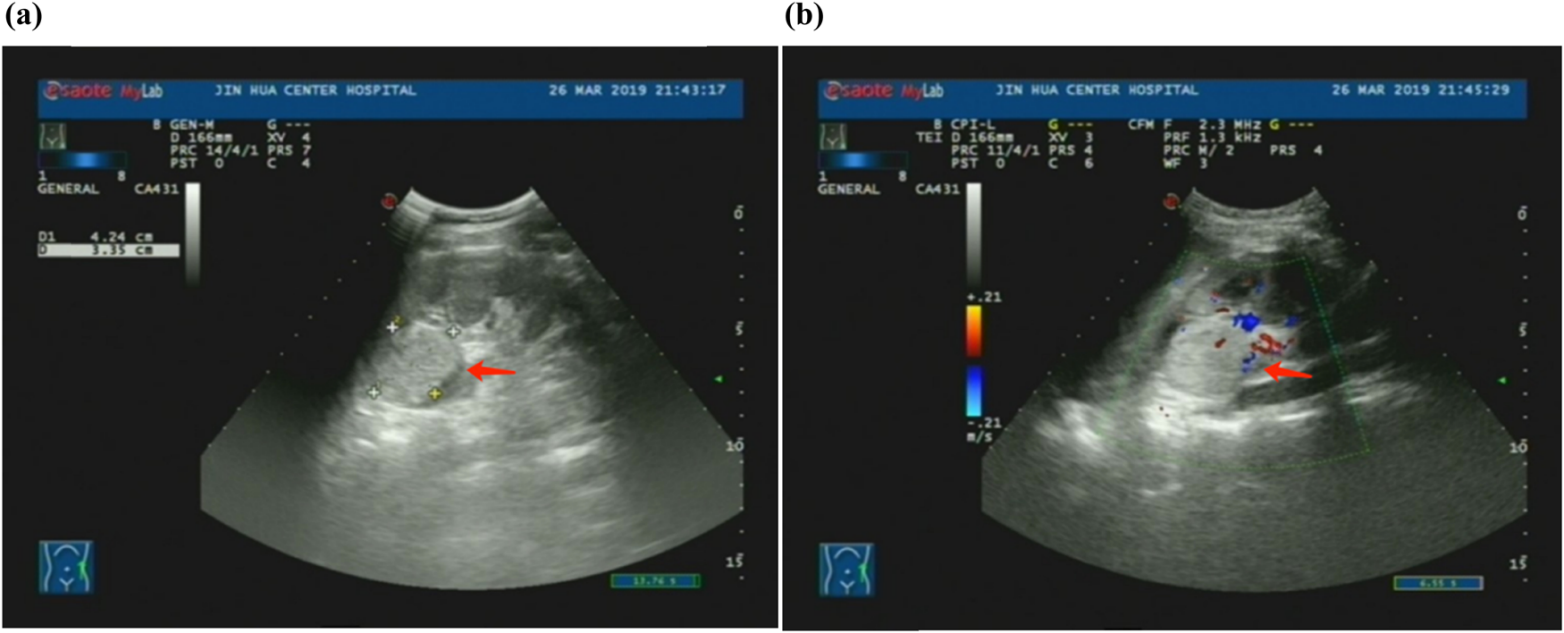
(a) Ultrasound showing a high echogenic area with internal uneven echo in the upper pole of the left kidney. (b) Ultrasound showing blood flow signal inside the echogenicity.

**Figure 3:**
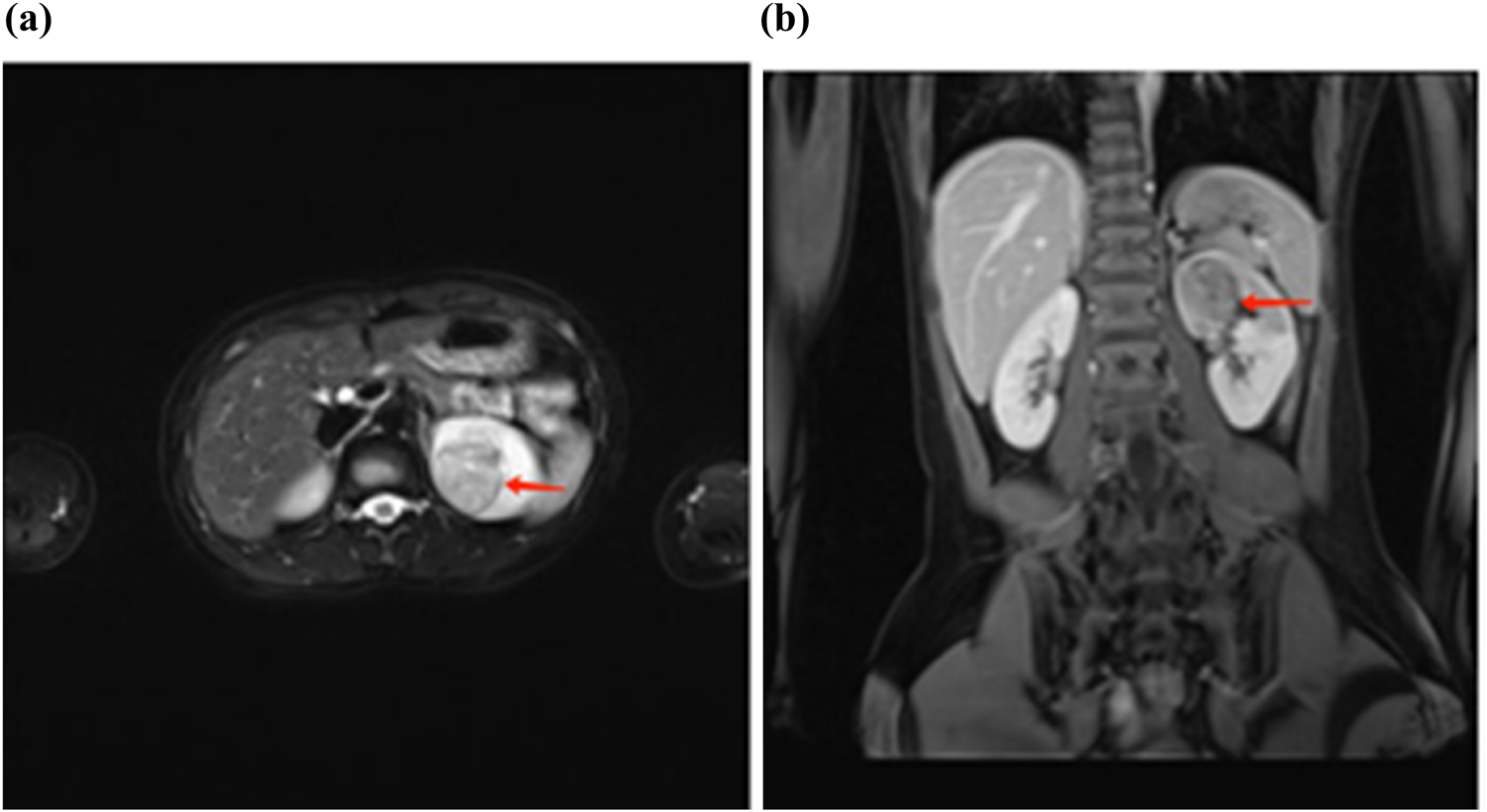
MRI image showing a solid mass in the upper pole of the kidney with long signal in T2WI: (a) transverse plane and (b) coronal plane.

A 16-year-old female patient presented at the urology clinic on March 25, 2019, with a complaint of hematuria lasting for 5 days. The patient was admitted to the hospital to identify the cause of the hematuria. Enhanced computed tomography (eCT) showed the presence of a solid mass (3.2 cm × 3.1 cm) in the dorsal upper pole of the left kidney, with evidence of invasion into the upper renal calyces (Figure 1). These findings were confirmed by ultrasonography (Figure 2) and enhanced magnetic resonance imaging (MRI) (Figure 3). Despite the apparent indications for surgical intervention, the patient’s guardians opted for Chinese Traditional Medicine (TCM) as an alternative treatment for the tumor, refusing surgical intervention. The patient was discharged following comprehensive communication and the completion of a consent form declining surgery.

**Figure 4:**
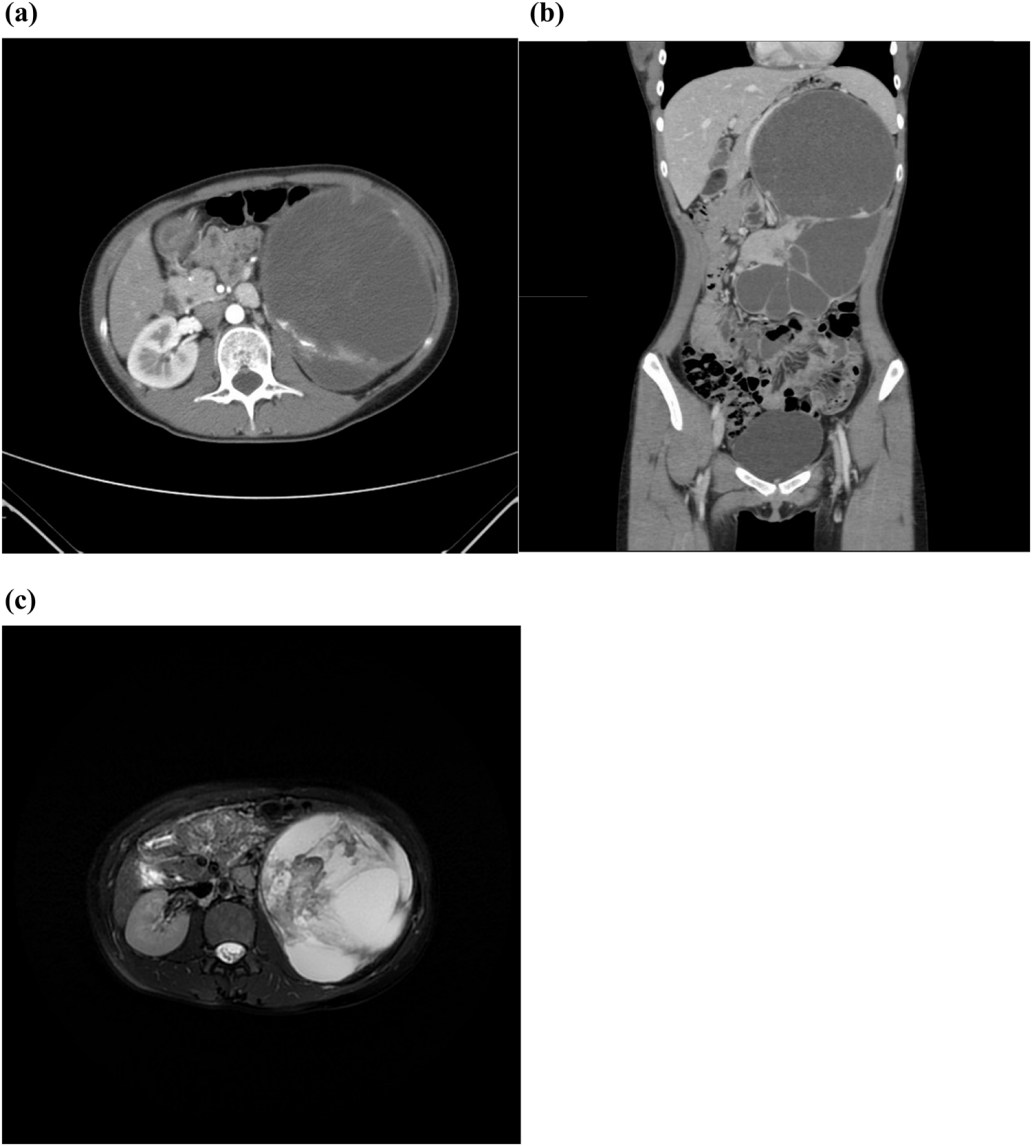
A sizeable cystic kidney with increased compartments was detected in the left abdomen on eCT and MRI scanning in 2023: (a) transverse plane, (b) coronal plane, and (c) T2 scan.

Four years later, on May 23, 2023, the patient, now 20 years old, revisited the Clinic of Urology with the complaint of a large mass in her left abdomen. Following admission to hospital, both eCT and enhanced MRI revealed the presence of left renal carcinoma with cystic changes, which had advanced considerably since March 30, 2019. The scans also detected invasion of the left calyces with left hydronephrosis. Furthermore, metastasis was indicated based on the observed enlargement of the retroperitoneal lymph nodes (Figure 4). The left renal glomerular filtration rate (GFR) was severely impaired, with GFR = 12.17 mL/min, while the right GFR was normal at 65.23 mL/min. No contraindications were identified during the preoperative evaluations. A palpable hard mass in the left upper abdomen was found during the physical examination.

**Figure 5:**
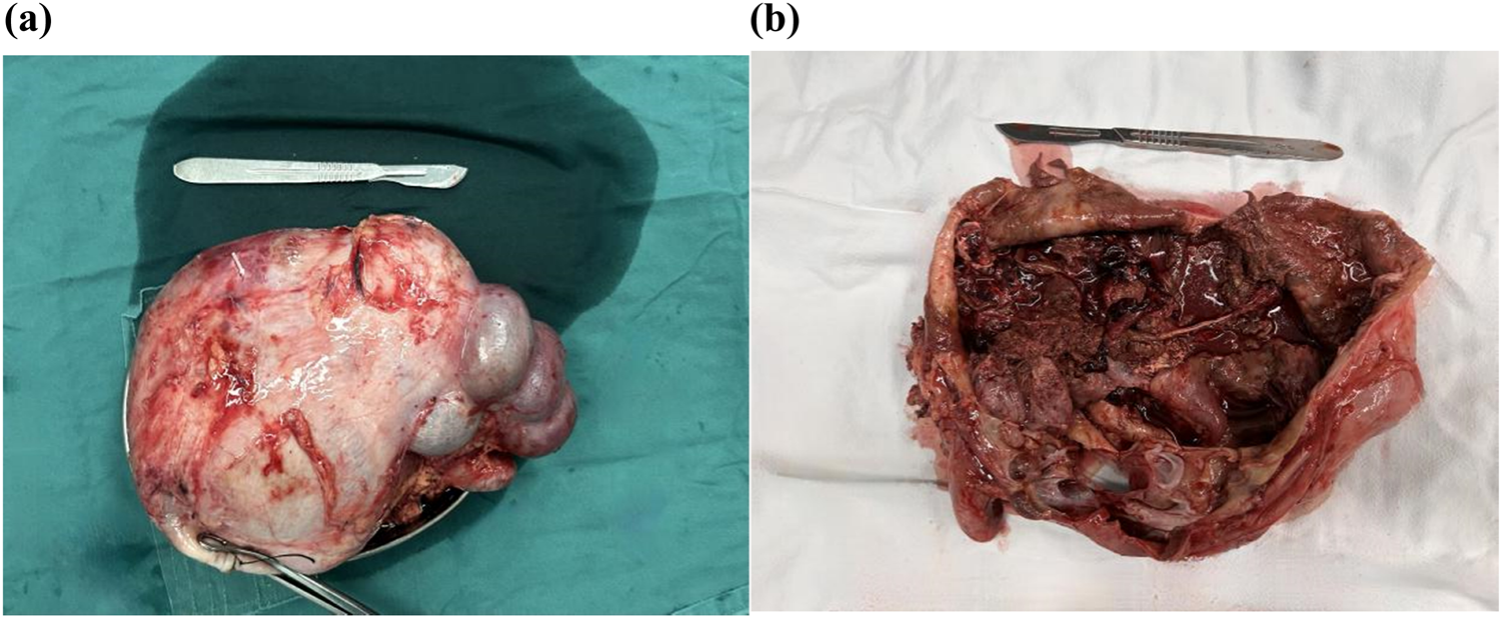
Postoperative gross morphology of a typical Xp11.2 translocation RCC. (a) Specimen obtained on RN, showing multiple protruding masses. (b) Dissected specimen showing the cystic tumor covering the whole kidney.

To ensure the successful completion of the operation, a multidisciplinary discussion was convened, and it was decided that retroperitoneal laparoscopy for left radical nephrectomy (RN) was the most effective approach. The patient provided written consent and underwent retroperitoneal lymph node dissection and radical resection of the left carcinoma under general anesthesia (Figure 5). Following a week of recovery from surgery, the patient was discharged from the hospital.

Postoperative pathology identified RCC measuring 12 × 7.5 × 5.5 cm, together with intravascular cancer thrombus, renal sinus invasion, and necrosis associated with Xp11.2 translocations/*TFE3* gene fusions.

In addition, analysis of the retroperitoneal lymph nodes (3/7) and hilar lymph nodes (11/20) revealed metastatic RCC. Based on the staging criteria of the American Joint Committee on Cancer (AJCC) (2017), the TNM staging was reclassified. The negative margin of pT3aN1M0 determined the pathological stage.

**Figure 6:**
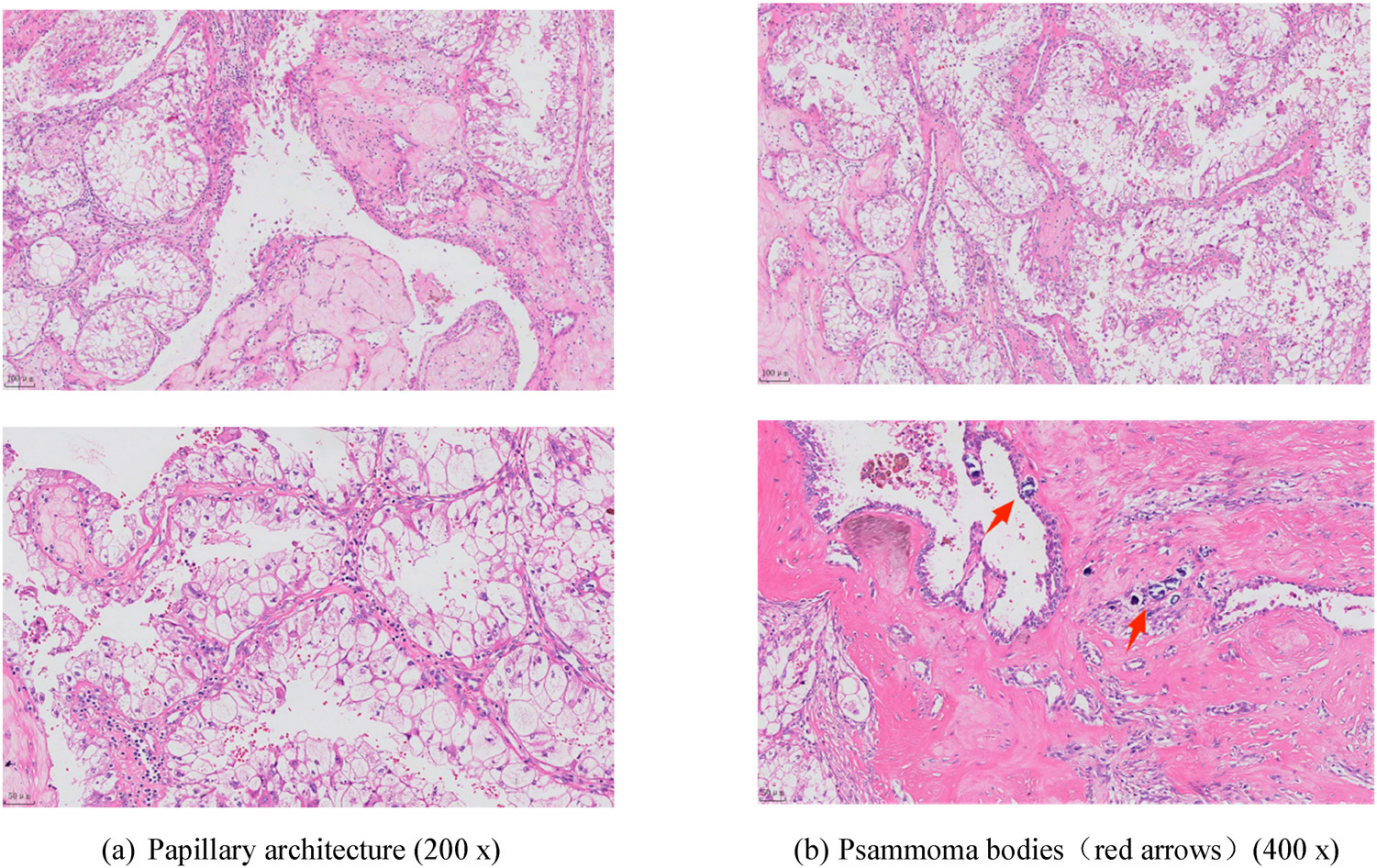
Microscopic examination of tumor cells with dense clear or eosinophilic cytoplasm and papillary and solid multilayered architecture. Hematoxylin and eosin (H&E) staining at 100× magnification. (a) Papillary architecture (200×) and (b) psammoma bodies (red arrows) (400×).

**Figure 7:**
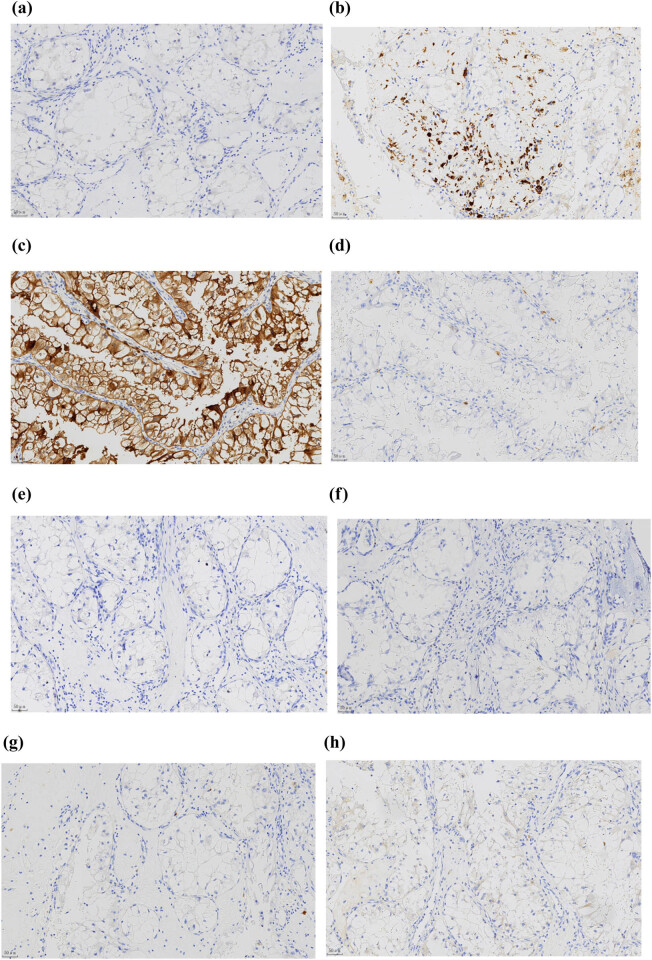

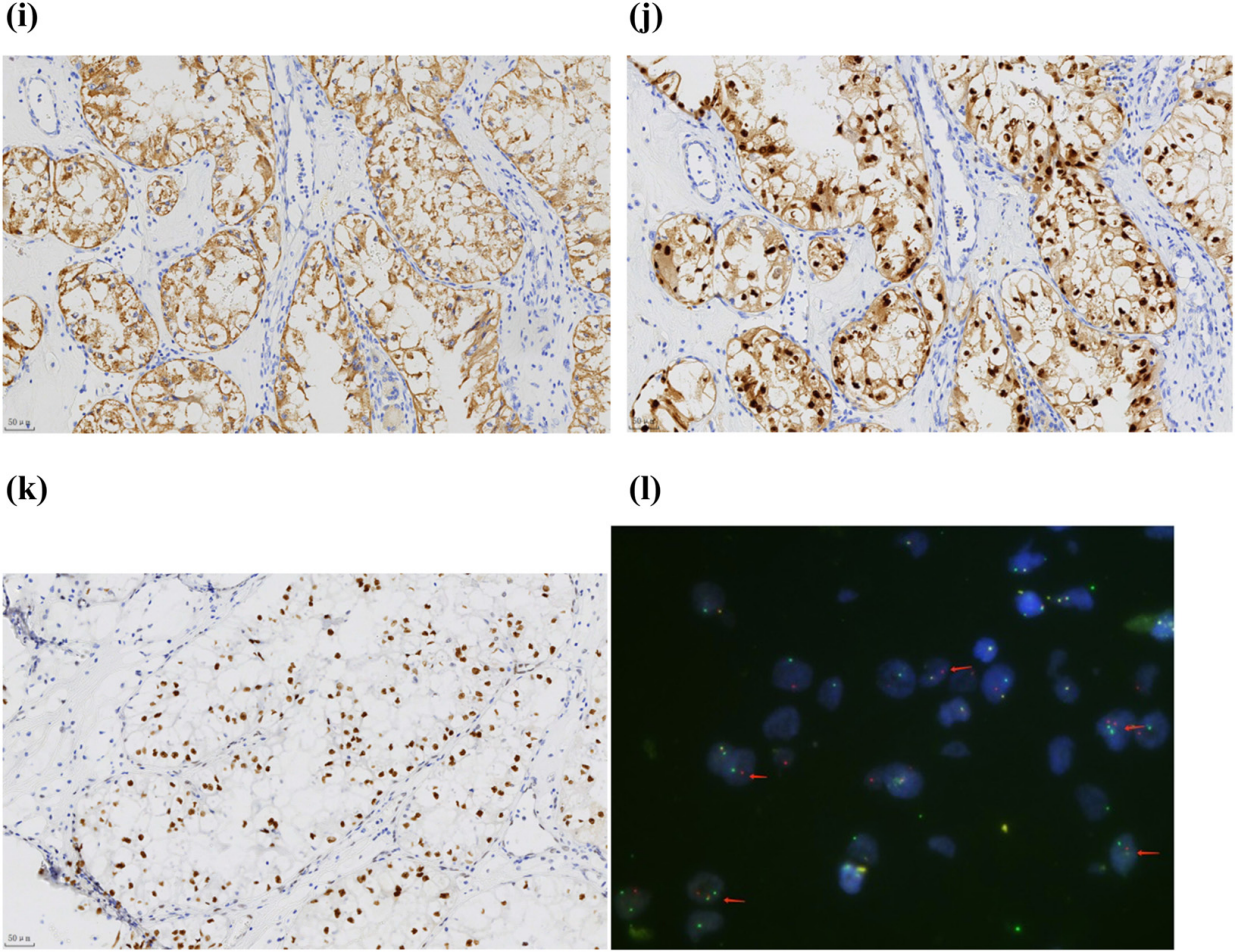
Immunohistochemical examination of the tumor cells (200× magnification) and FISH. The tumor cells were negative for CA IX (a), CD117 (d), CK7 (e), GATA3 (f), HMB45 (g), and melan-A (h) expression, using the EnVision two‐step immunostaining method at a magnification of 200×. Conversely, positive immunostaining was observed for CD10 (c), P504S (i), and PAX8 (j) using the same method and magnification. Additionally, the tumor cells displayed strong nuclear positivity for Cathepsin K (b) using the EnVision two‐step method and for TFE3 (k) using the SP method, both at a magnification of 200×. The TFE3 break-apart FISH assay (l) showed split green and red signals, indicating *TFE3* gene rearrangement.

Positive results were observed for CD10, AE1/AE3, Vimentin, Melan-A, P504 s, and *TFE3* via immunohistochemistry and fluorescence *in situ* hybridization (FISH) (Figures 6 and 7).

Immunoreactivity of nuclear *TFE3* indicated robust CD10, Cathepsin K, P504S, and PAX8 expression in the tumor. There was no staining for CA IX, CD117, CK7, and GATA3, nor for melanocytic markers (melan-A and HMB45).

Due to socioeconomic considerations, the patient received only TCM as treatment after surgery. No evidence of recurrence was seen at the 5-month follow-up after surgery. However, long-term follow-up is necessary to evaluate the patient’s mid-to-long-term prognosis. Given the staging of clinical stage III, according to the 2022 EAU guidelines on RCC [5], we recommend that the follow-up plan be followed strictly. Physical re-examination should be carried out every 3 months for 2 years, semiannually for 1 year, annually for 1 years, and biennially after 5 years. The physical re-examination includes physical examination, laboratory examination, abdominal ultrasound, chest CT, and abdominal CT.


**Informed consent:** Informed consent has been obtained from the patient.

## 3 Discussion

The well-known clear cell cancer represents the majority (80–85%) of all RCC cases. A specific type of RCC, Xp11.2-translocation RCC, is typically found in children (incidence of 20–40%), with a lower incidence in adults (1–1.6%). However, its one-third incidence is much higher in juveniles than in adults (0.2–5.0%) [6–9]. In 2016, the WHO introduced a new molecular-driven histotype classification, resulting in the classification of this tumor as MiT-RCC, indicating miT family translocation RCC [10].

The translocation of Xp11.2 RCC is distinguished by a gene fusion between *TFE3* and several other genes, including *ASPL* (17q25), *CLTC* (17q23), *NonO* (Xp12), *PSF* (1q34), and *PRCC* (1q21), along with the translocation on chromosome Xp11.2 [11].

RCCs resulting from Xp11.2 translocations are typically associated with advanced tumor stage and poor oncological prognosis due to their aggressive biological behavior. Pediatric patients with metastases have an average survival duration of 6.3 years, compared to the 2-year survival rate observed in adult patients [12]. A study conducted by Zhou et al. found that the overall survival (OS) rate closely matched the 5-year OS of RCCs. The study included 46 patients with Xp11.2-translocation RCC, and their OS rates were 97.4% at 1 year, 88.8% at 3 years, and 88.8% at 5 years. The increased proportion of cT1 Xp11.2 tumors in the group was then analyzed as a possible solution. This finding may also suggest that early treatment of Xp11.2-translocation RCCs (cT1∼2) may be related to a more favorable prognosis compared to patients who receive late treatment. Distal or regional lymph node metastasis may result in clinically unfavorable outcomes [13]. Despite its uncommon occurrence in adults, chemotherapy has the potential to cause chronic renal failure, which can lead to Xp 11.2 translocation of RCC.

Previous studies have indicated that children tend to have a more favorable prognosis than adults [3]. However, there is ongoing debate surrounding the prognosis of Xp11.2-translocation RCC. In contrast, Cheng et al. in a recent meta-analysis of 15 studies that examined 147 patients with Xp11.2-translocation RCC found no significant difference in prognosis between children and adults, males and females [4].

RCC with Xp11.2 translocation/*TFE3* gene fusion affects mostly adolescents, and no difference between the sexes was found [1]. However, several studies, such as that of Liu et al., observed a female predominance as females possess two X chromosomes. In contrast, males only have one, which results in a greater incidence of X chromosome translocation. These authors concluded by stating that due to the small sample size, further investigation was necessary to determine the exact frequency and mechanisms [14]. Compared to other subtypes of RCC, RCC caused by Xp11.2 translocations is more aggressive, with enhanced progression of local lesions and an increased risk for distant and lymphatic metastases. Moreover, its resistance to chemotherapy and radiotherapy ultimately results in a less favorable prognosis [1,2].

Most patients do not present with the renal cancer triad, which consists of gross hematuria, lumbago, and abdominal mass. Similar to other forms of RCC, nearly all patients with Xpl1.2 RCC do not exhibit the typical triad of renal cancer symptoms. Instead, they may only experience one of the three syndromes or even have no sign of noticeable clinical manifestations, making accurate diagnosis difficult. The most prevalent clinical manifestation of the renal triad is gross hematuria, as was found in the current case study.

Renal neoplasms are typically detectable via abdominal ultrasonography, CT, and MRI examinations. Each of the three methods possesses distinct advantages. However, abdominal ultrasonography is the most commonly used simple procedure for daily inspection, diagnosis, and postoperative assessments. Abdominal CT scans, particularly enhanced contrast CT scans, offer higher diagnostic sensitivity, specificity, and information regarding organs other than the kidney. Due to their non-radioactive nature, MRI examinations are safe for children and patients allergic to chemicals. Moreover, positron emission tomography-computed tomography aids in the diagnosis procedure and is helpful during the clinical stages.

Immunohistochemical staining of the *TFE3* gene fusion-associated protein is the primary diagnostic marker for Xp11.2 RCC [14]. In the current case study, the tumor was predominantly papillary in structure, and its histopathological analysis revealed cytoplasm-rich clear or eosinophilic epithelioid cells and numerous psammoma bodies.

Most cases are currently managed according to the treatment guidelines for conventional RCC, as standard treatment guidelines for Xp11.2 tRCC have not yet been published. Surgery is usually the most effective therapeutic approach for RCC, as the tumor is resistant to radiotherapy and chemotherapy. Patients with small lesions (≤7 cm) with distinct boundaries are advised to undergo nephron-sparing surgery (NSS), particularly those with an anatomical or functional solitary kidney, to allow retention of renal function. Based on the published literature, small diameters (≤7 cm) located in one pole and distinct rims in young patients are considered indicators for NSS [15,16]. Several studies have demonstrated that NSS yields positive treatment outcomes during short-term follow-up [17,18]. Considering the aggressive nature of the tumor as well as its tendency to invade nearby tissues, it is essential to consider the positive margins and the potential for lymphatic and distant organ metastasis. RN is critical in surgery for RCC where the diameter is greater than 7 cm, where there are unclear boundaries, or where the tumor cannot be completely removed via nephrectomy [19–21].

If the tumor size is around 7 cm, a comprehensive assessment should be made based on the location of the tumor. According to the RENAL score [22], the patient’s general condition, the technical level and experience of the surgeon, and the medical conditions of the hospital, are factors to consider when selecting the surgical approach.

A poor prognosis has been found to be associated with inferior vena cava tumor thrombosis and advanced TNM stage [15]. The examination of the young female patient in this case showed that she had a neoplasm in her left kidney, which was the probable cause of the gross hematuria. If the patient had not refused the treatment 4 years before, RN would have probably been sufficient. Unfortunately, after 4 years of natural rapid progression, the renal tumor had grown to 12 × 7.5 × 5.5 cm with cystic mass and necrosis. Additionally, the post-surgery pathology confirmed the presence of retroperitoneal lymph node enlargement, indicating metastasis.

The surgical procedure involved retroperitoneal laparoscopy for RN and lymph node dissection. The excision extension comprised various anatomical structures, including the ipsilateral adrenal gland, perirenal fascia, perirenal fat, and lymph nodes extending from the diaphragm’s crus to the abdominal aorta’s bifurcation. Based on the observed timeline, it is evident that Xp11.2-translocation RCC with *TFE3* gene fusion tumors are highly malignant and exhibit rapid progression.

RN is the first step for Xp11.2 RCC patients with lymphatic or distant organ metastases. To increase survival time, adjuvant treatment should be implemented; this may consist of immune checkpoint inhibitors (ICI) or targeted agents such sunitinib and sorafenib, although there are limited data on the use of these.

Previous studies have demonstrated that targeted agents can extend the survival of patients with Xp11.2-translocation RCC, lymph node metastasis, or distant metastasis without disease progression [23]. Based on the study conducted by Gao et al. and Masago et al., two ICIs, nivolumab and ipilimumab, were found to be clinically effective in patients with metastatic RCC [24,25]. Furthermore, a case presented by Zhao et al. demonstrated that a novel anti-PD-1 antibody (camrelizumab, 200 mg) combined with axitinib (5 mg) administered for 1 year was highly effective in treating an Xp11.2-RCC patient who had developed retroperitoneal lymph node metastases following surgery. The patient achieved a complete clinical response after 18 months of follow-up [26]. In a retrospective analysis by Boilève et al., the median progression-free survival for patients with translocation RCC during the first ICI treatment was only 2.5 months (range, 1–40 months), while four patients experienced a partial response (16.7%) and three (12.5%) had stable disease [27]. Yan et al. in a study of 40 patients who underwent first-line treatment mainly including sunitinib (*n* = 14), sorafenib (*n* = 15), axitinib (*n* = 6), and pazopanib (*n* = 5), the median progression-free survival of patients receiving these regimens were 7.4, 5.4, 9.4, and 8.9 months, respectively, which demonstrated some efficacy [28].

However, the effectiveness of adjuvant treatment is still a matter of debate. For instance, Masago et al. reported that immunotherapy consisting of nivolumab and ipilimumab failed to cure patients with metastatic Xp11.2 RCC [25]. Liu et al. found that all adults with stage III disease who received ORN/LRN and targeted molecular therapy subsequently progressed to develop terminal or recurrent cancer. The poor efficacy of drugs in improving patient outcomes is the first potential challenge. Second, drug side effects, such as proteinuria, hypertension, neutropenia, palmar-plantar erythrodysesthesia syndrome, and hypertriglyceridemia, should also be considered [29]. Third, some people cannot afford the high price. Therefore, the development of a reliable and efficient adjuvant treatment is still required.

Based on the current understanding, this was the first case report demonstrating the natural progression of a juvenile Xp11.2-translocation RCC with *TFE3* gene fusion and the catastrophic effects of neglecting the condition.

Collectively, juvenile Xp11.2-translocation RCC with *TFE3* gene fusion is a highly malignant form of the disease, characterized by rapid progression in the short term, resulting in distant metastasis and tumor enlargement. Proper attention needs to be directed to teenagers diagnosed with renal tumors. Before any other treatment approach, Xp11.2-RCC should be addressed with radical NSS or RN. Adjuvant therapy, consisting of targeted agents and ICI, could extend longevity. However, further investigation is urgently required to improve the efficacy of treatments for Xp11.2 RCC.
